# Contrasting Prefrontal Cortex Contributions to Episodic Memory Dysfunction in Behavioural Variant Frontotemporal Dementia and Alzheimer’s Disease

**DOI:** 10.1371/journal.pone.0087778

**Published:** 2014-02-04

**Authors:** Stephanie Wong, Emma Flanagan, Greg Savage, John R. Hodges, Michael Hornberger

**Affiliations:** 1 Neuroscience Research Australia, Randwick, Sydney, New South Wales, Australia; 2 Department of Psychology, Macquarie University, Sydney, New South Wales, Australia; 3 Australian Research Council Centre of Excellence in Cognition and its Disorders, Sydney, New South Wales, Australia; 4 School of Medical Sciences, University of New South Wales, Sydney, New South Wales, Australia; University of California, San Francisco, United States of America

## Abstract

Recent evidence has questioned the integrity of episodic memory in behavioural variant frontotemporal dementia (bvFTD), where recall performance is impaired to the same extent as in Alzheimer’s disease (AD). While these deficits appear to be mediated by divergent patterns of brain atrophy, there is evidence to suggest that certain prefrontal regions are implicated across both patient groups. In this study we sought to further elucidate the dorsolateral (DLPFC) and ventromedial (VMPFC) prefrontal contributions to episodic memory impairment in bvFTD and AD. Performance on episodic memory tasks and neuropsychological measures typically tapping into either DLPFC or VMPFC functions was assessed in 22 bvFTD, 32 AD patients and 35 age- and education-matched controls. Behaviourally, patient groups did not differ on measures of episodic memory recall or DLPFC-mediated executive functions. BvFTD patients were significantly more impaired on measures of VMPFC-mediated executive functions. Composite measures of the recall, DLPFC and VMPFC task scores were covaried against the T1 MRI scans of all participants to identify regions of atrophy correlating with performance on these tasks. Imaging analysis showed that impaired recall performance is associated with divergent patterns of PFC atrophy in bvFTD and AD. Whereas in bvFTD, PFC atrophy covariates for recall encompassed both DLPFC and VMPFC regions, only the DLPFC was implicated in AD. Our results suggest that episodic memory deficits in bvFTD and AD are underpinned by divergent prefrontal mechanisms. Moreover, we argue that these differences are not adequately captured by existing neuropsychological measures.

## Introduction

Behavioural variant frontotemporal dementia (bvFTD) is the second leading cause of early-onset dementia, after Alzheimer’s disease (AD) [1], [2]. Patients with bvFTD present with a range of symptoms, notably decline in social behaviour and personal conduct, ritualized activity, loss of empathy, emotional blunting and executive dysfunction [3]. While episodic memory deficits are a well-established early feature of AD [4], the diagnostic criteria for bvFTD mandate a predominantly dysexecutive cognitive profile, with relative sparing of episodic memory and visuospatial skills [3]. Indeed, an amnestic presentation still remains an exclusion criterion for diagnosis of bvFTD [3], [5].

Increasing evidence, however, shows that a proportion of bvFTD cases, including those with pathological confirmation, can present with marked episodic memory deficits [6]–[9], and are generally impaired on standard recall based memory tasks [10], despite relatively intact recognition memory compared to age-matched controls [11]–[13]. While some studies report greater impairment on measures of memory recall in AD compared to bvFTD [14]–[16], others have demonstrated that patients with bvFTD show comparable deficits [17]–[20]. The reason for these discrepant results is currently unclear, but may be due to various factors, such as disease progression, types of memory measures and the inclusion of the recently recognised non-progressive bvFTD ‘phenocopy’ patients [17].

Investigations into the underlying neural correlates of episodic memory deficits usually focus on medial temporal lobe (MTL) damage, particularly in the hippocampus. Episodic memory impairments in AD have largely been attributed to hippocampal atrophy [18], [21]. Not surprisingly, in light of the recent memory findings, bvFTD patients show similar degrees of hippocampal atrophy during earlier disease stages compared to AD [18], [22], and this can be even more severe in bvFTD at post mortem [23]. Nevertheless, the extent to which the pervasive prefrontal cortex atrophy in bvFTD contributes to their amnesia is unclear. Indeed, evidence from visual atrophy rating [18], whole-brain voxel-based morphometry (VBM) [24] and 18F-fluorodeoxyglucose positron emission tomography (FDG-PET) [19] studies, in which the degree of episodic memory deficits was covaried with brain dysfunction, show that not only MTL but also prefrontal atrophy contribute to the episodic memory deficits in bvFTD.

The role of the prefrontal cortex (PFC) in episodic memory is still controversial [25]. Current evidence from neuroimaging and lesion studies suggests that the strategic aspects of episodic memory recall are mediated by PFC structures [26], [27], in particular the dorsolateral PFC (DLPFC) [28], [29]. Accordingly, it has been proposed that episodic memory deficits in bvFTD may be related to failure of strategic retrieval processes due to difficulties with planning and organisation of information during encoding and/or retrieval [30]. Further support for this arises from studies that have demonstrated associations between impaired autobiographical memory retrieval and executive dysfunction in bvFTD [31]. Executive dysfunction is also a prominent component of AD [4], [32], however, and is associated with memory deficits [33] and PFC atrophy [34]. DLPFC atrophy is evident in both AD and bvFTD and does not serve as a reliable marker to distinguish between the two diseases [35]. This raises the question as to whether other PFC regions might contribute to the memory deficits seen in bvFTD.

The ventromedial prefrontal cortex (VMPFC) emerges as the region which most likely influences episodic memory performance in bvFTD because it is affected very early in the illness [35]–[37] and shows strong connections with the MTL [38]. Very few studies, however, have investigated the relative contribution of VMPFC dysfunction to episodic memory recall in bvFTD. For example, Pennington and colleagues [18] revealed that correlations between PFC atrophy and episodic memory deficits were strongest for the VMPFC and not DLPFC. Similarly, impaired autobiographical memory recall appears to be related to VMPFC dysfunction [39]. This is corroborated by functional imaging studies in healthy participants, which have shown that contextual information retrieval is associated with MTL-VMPFC interaction [40], [41]. Still, to date no study has directly contrasted the DLPFC and VMPFC contributions to episodic memory deficits in bvFTD and AD to reveal such a dissociation.

The current study set out to address this issue by directly contrasting DLPFC and VMPFC functions and their contributions to episodic memory in bvFTD and AD. In particular, we employed neuropsychological measures typically tapping into either DLPFC or VMPFC functions, to quantify the relationship between performance on these tasks and measures of episodic memory recall. We further sought to elucidate the prefrontal neural substrates of these relationships using VBM covariate analyses. Based on previous evidence we predicted that episodic memory dysfunction in both patient groups would relate to divergent patterns of prefrontally mediated task performance and grey matter atrophy. Specifically, in bvFTD we hypothesised that episodic memory impairment would be mainly related to VMPFC-mediated tasks and atrophy. In contrast, we predicted that episodic memory impairment in AD would be more correlated with DLPFC-mediated tasks and atrophy.

## Methods

### Case Selection

A sample of 22 bvFTD and 32 AD patients and 35 age- and education-matched controls were selected from the FRONTIER database, resulting in a total of 89 participants. All bvFTD patients fulfilled proposed criteria for possible bvFTD [3] as well as consensus criteria for FTD [5], with insidious onset, decline in social behaviour and personal conduct, emotional blunting and loss of insight. All AD patients met NINCDS-ADRDA diagnostic criteria for probable AD [4]. Disease duration was estimated as the number of years elapsed since onset of symptoms. The age- and education-matched healthy control group consisted of volunteers or spouses/carers of patients (see Table 1 for demographic details). To determine their overall level of cognitive functioning, all participants underwent general cognitive screening using the Addenbrooke’s Cognitive Examination-Revised (ACE-R) [42]. The Frontotemporal Dementia Rating Scale (FRS) [43] and Clinical Dementia Rating Scale (CDR) [44] were used to determine the disease severity in bvFTD and AD patients. In addition, the Cambridge Behavioural Inventory revised (CBI-R) [45] was used to quantify symptoms of behavioural disturbance reported by the family or carer, with higher scores indicative of more behavioural disturbance.

**Table 1:** Demographic characteristics and experimental composite scores across participant groups^a^.

	*Controls*	*bvFTD*	*AD*	*Group* *effect*	*bvFTD vs. Control*	*AD vs. Control*	*bvFTD vs. AD*
**N**	35	22	32				
**Sex (M/F)**	19/16	14/8	20/12	n.s.	.	.	.
**Mean age (years)**	64.20 (5.38)	61.23 (7.45)	63.53 (6.98)	n.s.	.	.	.
**Education (years)**	12.79 (2.60)	11.33 (2.51)	12.41 (3.28)	n.s.	.	.	.
**Disease duration (years)**	.	3.82 (2.44)	3.20 (2.09)	.	.	.	n.s.
**FRS Rasch score** b	.	−0.37 (1.02)	0.25 (0.96)	.	.	.	n.s.
**CDR sum of boxes ** [**18**] b	0.32 (0.46)	7.08 (2.86)	5.05 (2.68)	***	***	***	**
**ACE-R [100]**	95.00 (3.3)	76.00 (10.47)	67.84 (17.75)	***	***	***	n.s.
**CBI-R total frequency score [180]**	3.77 (4.57)	67.36 (34.72)	40.33 (26.48)	***	***	***	n.s.
**CBI-R selected subscores:**							
Memory/Orientation [32]	1.50 (2.68)	16.18 (6.95)	16.00 (7.27)	***	***	***	n.s.
Everyday skills [20]	0.17 (0.389)	7.09 (5.49)	7.25 (5.599)	***	***	***	n.s.
Abnormal behaviour [24]	0.25 (0.45)	9.18 (6.74)	2.08 (2.31)	***	***	n.s.	**
Stereotypic/motor behaviours [16]	0.58 (0.79)	7 (5.33)	1.5 (2.15)	**	***	n.s.	**
**Recall composite**	99.56 (21.1)	37.86 (28.76)	20.55 (18.69)	***	***	***	n.s.
**DLPFC task composite**	99.53 (18.88)	54.60 (26.03)	57.39 (21.73)	***	***	***	n.s.
**VMPFC task composite**	99.81 (7.95)	69.26 (17.19)	82.88 (15.03)	***	***	***	***

a Standard deviations in parentheses, maximum score for tests shown in brackets.

b All patients had either FRS or CDR disease severity measures.

** *p*<.01, ***p<.001, n.s. = non-significant.

### Ethics Statement

All participants provided written informed consent, and dual consent was obtained from the carer for some participants. This study was approved by the South Eastern Sydney and Illawarra Area Health Service and the University of New South Wales ethics committees.

### Neuropsychological Measures

The Rey Auditory Verbal Learning Test (RAVLT) [46] was administered as a measure of episodic memory recall for verbal information. The RAVLT involves learning a list of 15 words (List A), which is read aloud over five consecutive trials, each followed by a free recall test. This is followed by presentation of an interference list of 15 words (List B), with a free recall test for these words. Participants are then required to recall words from List A without further presentation of those words. Following a 30-minute delay, recall of List A is re-assessed, followed by a recognition test, containing all items from List A as well as words from List B and 20 new words. The immediate recall following interference trial (A6) score was included in our analyses.

Episodic memory recall for visual information was assessed using the Rey-Osterrieth Complex Figure (RCF) test [47]. Three minutes after copying a complex figure as accurately as possible, participants were instructed to reproduce the figure from memory. The 3-minute recall score was included in our analyses.

The following measures of prefrontal function were administered: the Controlled Oral Word Association Test (COWAT) [48], the Backwards Digit Span test [49], the Brixton Spatial Anticipation Test and the Hayling Sentence Completion Test [50], the Iowa Gambling Task (IGT) [51] and The Awareness of Social Inference Test (TASIT) [52].

The COWAT is a timed task that involves generating a list of words that begin with a specified letter (over 3 trials, for F, A or S). The Backwards Digit Span is measure of working memory, where participants are required to repeat series of numbers (which increase in length over trials) in backwards order. In healthy adults, performance on the COWAT is associated with DLPFC grey matter volume [53] and activation of the DLPFC has been demonstrated during the Backwards Digit Span task [54]. The total correct scores were recorded for both the COWAT and Backwards Digit Span tests.

The Brixton Spatial Anticipation Task involves rule attainment and the use of feedback to guide future actions. In this task, participants view several pages with an array of ten circles. In each array, one of the ten circles is coloured blue and the position of the blue circle varies from page to page in accordance with simple rules. The participant is required to predict the location of the blue circle on subsequent pages, based on its location in previous pages. Although few studies have systematically examined the neural correlates that underpin performance on the Brixton Test, one study has found significantly impaired performance in patients with focal left lateral PFC lesions [55]. To allow comparison between neuropsychological measures, the Brixton total error score was converted to a total correct score.

The Hayling Test assesses the ability to inhibit prepotent verbal responses on a sentence completion task. An initial baseline phase requires completion of a sentence with a logical word as quickly as possible; the second phase involves inhibition of an automatic logical response, and rather, completion of the sentence with a word that is semantically unrelated. Performance on the Hayling Test is correlated with orbitofrontal cortex (OFC) atrophy in bvFTD [56]. The total number of errors scored by each participant on Section 2 of the test was subtracted from the maximum possible error score to allow comparison between neuropsychological measures, such that lower values indicate greater impairment.

The IGT is a computer-administered task, which involves selecting cards from four decks, each of which is associated with varying degrees of monetary profit or loss. Overall, selecting cards from decks A and B results in larger net loss, whereas selecting cards from decks C and D leads to greater net profit. The total number of cards chosen from each of the four decks was recorded, from which a modified total net score (decks D – A) was calculated. Positive scores indicate a dominance of advantageous deck choices, whereas negative scores indicate a dominance of disadvantageous deck choices. IGT task performance in bvFTD is correlated with VMPFC atrophy [57]. To allow conversion of IGT scores into percentages of the control mean for calculation of composite scores, scores were linearly transformed to ensure all scores were positive.

The Emotion Evaluation subtest from the TASIT evaluates comprehension of basic emotion through 28 professionally enacted video vignettes, portraying positive (happiness, surprise or neutral) or negative (sadness, anger, anxiety or revulsion) emotions. Participants are shown a response card listing each of the emotions in a random order, and are required to state the emotion that is being portrayed by the actor. In bvFTD patients, poor negative emotion recognition is associated with OFC atrophy [58]. The total number of correct responses was recorded.

### Composite Scores

All neuropsychological test scores were converted into percentage of the control mean, before averaging to yield composite scores. RAVLT and RCF recall scores were averaged to produce a recall composite. Based on previous studies that demonstrate associations between task performance and regional atrophy or activation, prefrontal tasks were subdivided into DLPFC task and VMPFC task composite scores. The DLPFC task composite included scores from the COWAT, Backwards Digit Span and Brixton Spatial Anticipation tasks. Scores from the Hayling Sentence Completion Task, IGT and TASIT were included in the VMPFC task composite score.

### Statistics

Data were analysed using SPSS20.0 (SPSS Inc., Chicago, Ill., USA). Kolmogorov-Smirnov tests were used to check for normality of distribution in the demographic data, neuropsychological measures and composite scores. Where the data were normally distributed, scores were compared across the three groups (bvFTD, AD and controls) using ANOVAs followed by Bonferroni *post-hoc* tests. Data that were not normally distributed were analysed using Kruskal-Wallis tests followed by *post-hoc* Mann-Whitney U tests with Bonferroni correction for multiple comparisons. A chi-square test was used to check for significant gender differences across groups.

### Image Acquisition and Voxel-based Morphometry (VBM) Analysis

All patients and controls underwent the same imaging protocol with whole-brain T1-weighted images using a 3T Phillips MRI scanner with a standard quadrature head coil (8 channels). The 3D T1-weighted sequences were acquired as follows: coronal orientation, matrix 256×256, 200 slices, 1×1 mm^2^ in-plane resolution, slice thickness 1 mm, TE/TR = 2.6/5.8 ms. 3D T1-weighted sequences were analysed using FSL-VBM, a voxel-based morphometry analysis [59], [60], which is part of the FLS software package http://www.fmrib.ox.ac.uk/fsl/fslvbm/index.html [61]. Following brain extraction from the images, tissue segmentation was carried out using FMRIB’s Automatic Segmentation Tool (FAST) [62]. The resulting gray matter partial volume maps were aligned to the Montreal Neurological Institute standard space (MNI52) using the nonlinear registration approach with FNIRT [63], [64], which uses a b-spline representation of the registration warp field [65]. To correct for local expansion or contraction, the registered partial volume maps were modulated by dividing them by the Jacobian of the warp field. The modulated images were then smoothed with an isotropic Gaussian kernel with a standard deviation of 3 mm (FWHM: 8 mm). Next, a voxelwise general linear model (GLM) was applied and permutation-based non-parametric testing (with 500 permutations per contrast) was used to form clusters with the Threshold Free Cluster Enhancement (TFCE) method [66]. Given our focus on the PFC and MTL involvement in memory recall, the VBM analysis was limited to the temporal and frontal lobes by creating a mask using the Montreal Neurological Institute standard space (MNI152) atlas. Based on the a priori hypothesis that performance on tasks included in the DLPFC task composite is related to the integrity of the DLPFC, VBM analysis of this composite score was performed using a mask including this region. Similarly, VBM analysis of the VMPFC task composite was limited to the VMPFC by using a mask for this region.

Group comparisons and covariate analyses of the composite data were tested for significance at *p*<.05, corrected for multiple comparisons via Family-wise Error (FWE) correction across space. Within patient groups, covariate analyses were conducted at significance levels of *p*<.05, False Discovery Rate (FDR) corrected. This increases sensitivity by controlling the expected proportion of false positives among suprathreshold voxels only, rather than all false positives across all voxels. Regions of significant atrophy were superimposed on T1-weighted standard brain images for spatial normalization and visual comparison with a brain atlas, allowing localisation of areas of significant grey matter loss. A cluster threshold of 50 contiguous voxels for significant atrophy clusters was applied and regions of atrophy are reported in MNI coordinates. To increase sensitivity, the cluster threshold was lowered to 20 contiguous voxels for within patient group analyses.

## Results

### Demographics and Global Cognitive Functioning

Demographics and general cognitive scores can be seen in Table 1. As a Bonferroni correction was applied, all *post hoc* group comparisons are reported at a.0167 level of significance. Participant groups did not differ in terms of age, sex or education (*p’s>*.1). The bvFTD and AD patient groups were matched for disease duration (*p*>.1). While disease severity did not differ between patient groups on the FRS (*p*>.05), the mean CDR sum of boxes score was higher in bvFTD compared to AD patients (*p* = .008). On the cognitive screening test (ACE-R), both patient groups were significantly impaired in comparison to controls (*p*<.001) but did not differ (*p*>.1). Based on CBI-R scores, both patient groups showed significantly more symptoms of overall behavioural disturbance compared to age-matched controls (*p’s<*.001), with a trend towards more severe symptoms in bvFTD compared to AD patients, though this did not survive correction for multiple comparisons (*p* = .039). Further analysis of selected CBI-R subscales showed that although memory/orientation and everyday skills (*p’s*>.05) were equally impaired in both patient groups, bvFTD patients showed more severe symptoms of abnormal behaviour and stereotypic/motor behaviours (*p’s*<.01).

### Neuropsychological Measures

The results of the neuropsychological measures are shown in Table S1. Distributions across all measures were non-normal, except for the COWAT. On measures of memory recall, both patient groups were significantly impaired compared to controls (*p’s*<.001). While RAVLT scores did not differ between patient groups (*p* = .238), there was a trend for better RCF recall performance in bvFTD compared to AD patients (*p* = .032), though this did not survive correction for multiple comparisons. On all measures of prefrontal function, both patient groups were significantly impaired in comparison to controls (*p’s*<.0167). However, bvFTD and AD patients only differed significantly on the Hayling AB error score (*p* = .008), with bvFTD patients making overall more errors.

### Composite Scores

Results for the composite scores are shown in Table 1. Distributions were normal for the DLPFC task and VMPFC task composites but non-normal for the recall composite. Both patient groups were significantly impaired across all composite scores, in comparison to controls (*p’s*<.001). Although there was a trend for worse recall performance in AD compared to bvFTD patients, this did not survive correction for multiple comparisons (*p* = .025). There were no significant differences between bvFTD and AD patients in terms of DLPFC task composite scores (*p*>.1). In contrast, VMPFC task composite scores were significantly lower in bvFTD patients compared to AD patients (*p* = .001).

### Correlations between Composite Scores

Spearman rank correlations were used to quantify relationships between the composite scores. Across all groups, the recall composite was significantly correlated with both the DLPFC task (r_s_ = .589, *p*<.001) and VMPFC task (r_s_ = .558, *p*<.001) composites. Correlations between composite scores failed to reach statistical significance in either patient group, separately.

### VBM Results

#### Group analysis

Participant groups were contrasted to reveal patterns of brain atrophy in the frontal and temporal mask. In comparison to controls, bvFTD patients showed widespread atrophy in frontal polar, orbitofrontal, anterior temporal, hippocampal, paracingulate and insular regions (Figure S1A). For AD patients, significant atrophy was found in comparison to controls, encompassing hippocampal, temporal, paracingulate and frontal regions (Figure S1B). Direct comparison of patient groups revealed significantly greater atrophy of the prefrontal and anterior temporal regions in bvFTD (Figure S1C). The reverse contrast did not reveal any regions of significantly greater atrophy in AD compared to bvFTD (Figure S1D).

#### Correlations with composite scores across all participants

**Figure 1:**
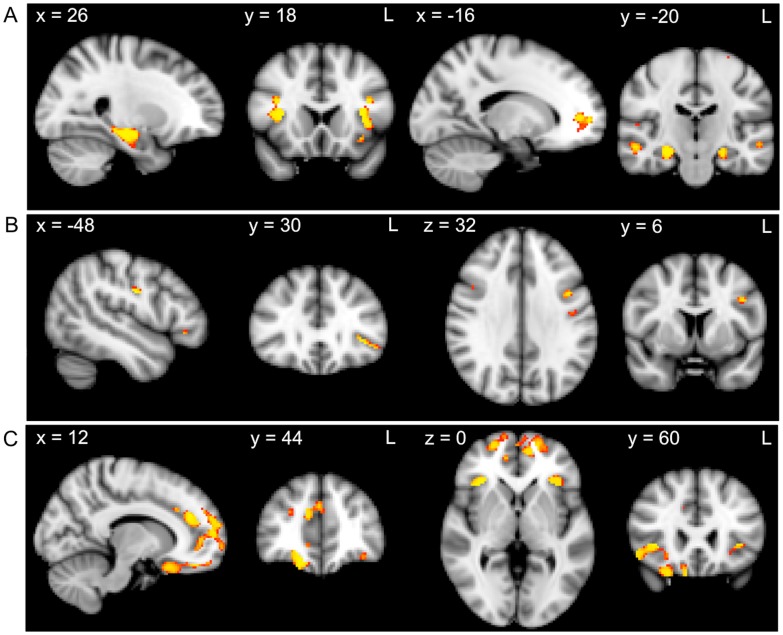
Grey matter atrophy correlates for recall, DLPFC task and VMPFC task performance across all participants. VBM analysis showing brain regions in which grey matter intensity correlates with the A) recall composite, B) DLPFC task composite and C) VMPFC task composite. Clusters are overlaid on the MNI standard brain. Coloured voxels show regions that were significant in the analysis for *p*<.05, corrected for multiple comparisons via Family-wise Error correction across space, and a cluster threshold of 50 contiguous voxels.

Composite scores were entered as covariates in the design matrix of the VBM analysis. FWE corrected significance levels of *p*<.05 and a cluster threshold of 50 contiguous voxels was used for all significant atrophy clusters. Across all participants, regions of atrophy that correlated with the recall composite included the insular cortex, frontal operculum cortex, middle and inferior temporal gyri, parahippocampal gyrus, hippocampus, frontal pole and temporal fusiform gyrus (Figure 1A, Table 2). The DLPFC task composite correlated with atrophy in the pre- and post-central gyri and the inferior and middle frontal gyri (Figure 1B, Table 2), whereas the VMPFC task composite was correlated with frontal pole, frontal operculum cortex, orbitofrontal cortex, paracingulate gyrus and insular cortex atrophy (Figure 1C, Table 2).

**Table 2:** Voxel-based morphometry results showing regions of significant grey matter intensity decrease that covary with composite scores across all groups.

		MNI Coordinates				
Regions	Hemisphere(L/R/B)	X	Y	Z	Number of voxels	T-score (peak voxel)
***Recall***						
Insula cortex/frontal operculum cortex	L	−38	20	0	395	2.96
Middle temporal gyrus	R	58	−32	−4	292	2.96
Parahippocampal gyrus	R	26	−20	−18	285	2.96
Hippocampus	L	−24	−14	−20	268	2.96
Frontal operculum cortex/insula cortex	R	36	18	8	220	2.96
Frontal pole	L	−16	56	2	172	2.96
Temporal fusiform cortex/parahippocampal gyrus	L	−30	−14	−36	99	2.96
Inferior temporal gyrus	R	50	−42	−18	58	2.46
***DLPFC tasks***						
Postcentral gyrus/precentral gyrus	L	−48	−10	30	52	2.96
Postcentral gyrus	L	−30	−28	62	27	2.96
Middle frontal gyrus	L	−42	6	32	23	2.71
Inferior frontal gyrus	L	−42	30	−2	22	2.71
***VMPFC tasks***						
Frontal pole	R	18	44	−22	1260	2.96
Frontal operculum cortex/orbitofrontal cortex	R	36	24	0	726	2.96
Paracingulate gyrus	L	−10	54	6	486	2.71
Paracingulate gyrus	R	12	38	22	176	2.96
Insular cortex/orbitofrontal cortex	L	−32	26	2	102	2.71
Frontal pole/orbitofrontal cortex	L	−34	40	−16	73	2.57

All results corrected at *p*<.05; only clusters with at least 50 contiguous voxels included.

#### Correlations with composite scores within patient groups

**Figure 2:**
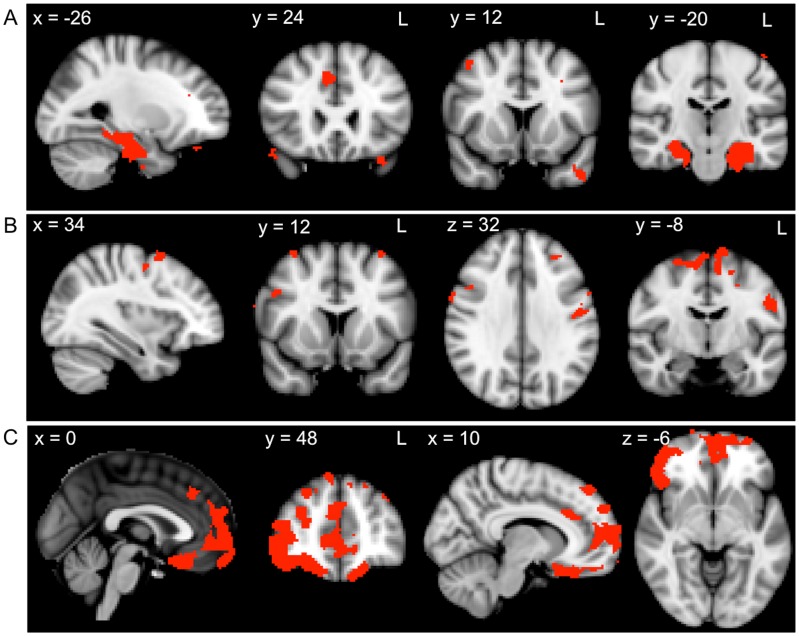
Grey matter atrophy correlates for recall, DLPFC task and VMPFC task performance within the bvFTD group. VBM analyses showing brain regions in which grey matter intensity correlates with the A) recall composite, B) DLPFC task composite and C) VMPFC task composite in bvFTD patients. Clusters are overlaid on the MNI standard brain. Coloured voxels show regions that were significant in the analyses for *p*<.05 FDR corrected and a cluster threshold of 20 contiguous voxels.

In further analyses, we investigated the correlations between the composite scores and regions of atrophy for each patient group. FDR corrected significance levels of *p*<.05 and a cluster threshold of 20 contiguous voxels were used for all significant atrophy clusters. In bvFTD patients, recall measures were associated with atrophy in regions including the parahippocampal gyrus, hippocampus, temporal pole, paracingulate gyrus, frontal pole, orbitofrontal gyrus, and superior and middle frontal gyri (Figure 2A, Table 3). Scores on the DLPFC task composite were related to atrophy in the middle and superior frontal gyrus, precentral gyrus, supplementary motor cortex, postcentral gyrus and posterior cingulate gyrus (Figure 2B, Table 3). In contrast, the VMPFC task composite was associated with atrophy in the orbitofrontal cortex, medial frontal cortex, anterior and paracingulate gyri, frontal pole and frontal operculum cortex (Figure 2C, Table 3).

**Table 3:** Voxel-based morphometry results showing regions of significant grey matter intensity decrease that covary with composite scores within the bvFTD group.

		MNI Coordinates				
Regions	Hemisphere (L/R/B)	X	Y	Z	Numberof voxels	T-score(peak voxel)
***Recall***						
Parahippocampal gyrus/hippocampus	L	−24	−20	−34	1068	3.23
Parahippocampal gyrus/hippocampus	R	22	−20	−32	813	3.23
Temporal pole	L	−48	18	−44	80	3.23
Paracingulate gyrus	R	6	24	34	53	3.23
Frontal pole	R	8	42	−28	48	3.23
Orbitofrontal cortex	L	−32	22	−26	38	3.23
Paracingulate gyrus	R	18	50	4	31	3.23
Frontal pole	R	42	60	6	29	3.23
Superior frontal gyrus	L	−22	8	72	27	3.23
Middle frontal gyrus	R	32	30	22	24	3.23
Frontal pole/Orbitofrontal cortex	L	−28	36	−24	24	3.23
***DLPFC tasks***						
Precentral gyrus/Supplementary cortex	R	6	−14	58	502	3.23
Precentral gyrus/Postcentral gyrus	L	−48	−10	28	108	3.23
Middle frontal gyrus	R	34	8	58	69	3.23
Precentral gyrus	R	16	−28	40	43	2.93
Middle frontal gyrus	L	−34	14	62	33	2.93
Frontal pole/Middle frontal gyrus	L	−28	38	30	31	3.23
Precentral gyrus/Superior frontal gyrus	L	−20	−14	56	29	3.23
Precentral gyrus	L	−45	−10	32	28	2.52
Precentral gyrus/Middle frontal gyrus	R	34	−6	52	27	3.23
Precentral gyrus	L	−57	4	38	25	2.75
Precentral gyrus	R	62	6	32	23	2.52
Superior frontal gyrus	R	12	4	72	22	2.75
Cingulate gyrus (posterior)	L	−14	−32	40	21	2.93
***VMPFC tasks***						
Orbitofrontal cortex/Medial prefrontal cortex/Frontal pole	B	12	24	−26	5589	3.23
Frontal pole	R	8	46	46	96	3.23
Anterior cingulate gyrus	R	10	24	28	86	2.93
Paracingulate gyrus	B	2	32	44	69	3.23
Frontal pole	R	24	46	38	62	2.75
Frontal pole	L	−52	42	12	57	3.23
Frontal pole	L	−38	44	36	46	3.23
Frontal pole/Superior frontal gyrus	L	−24	38	48	40	2.3
Frontal operculum cortex/Orbitofrontal cortex	L	−34	26	2	33	2.36
Anterior cingulate gyrus	L	−8	−14	38	28	2.93
Frontal pole	L	−12	52	44	25	2.52
Frontal pole	L	−26	38	28	24	2.3
Frontal pole	L	−18	64	26	22	2.3
Orbitofrontal cortex	R	26	24	−26	21	2.1

Results FDR corrected at *p*<.05; only clusters with at least 20 contiguous voxels included.

**Figure 3:**
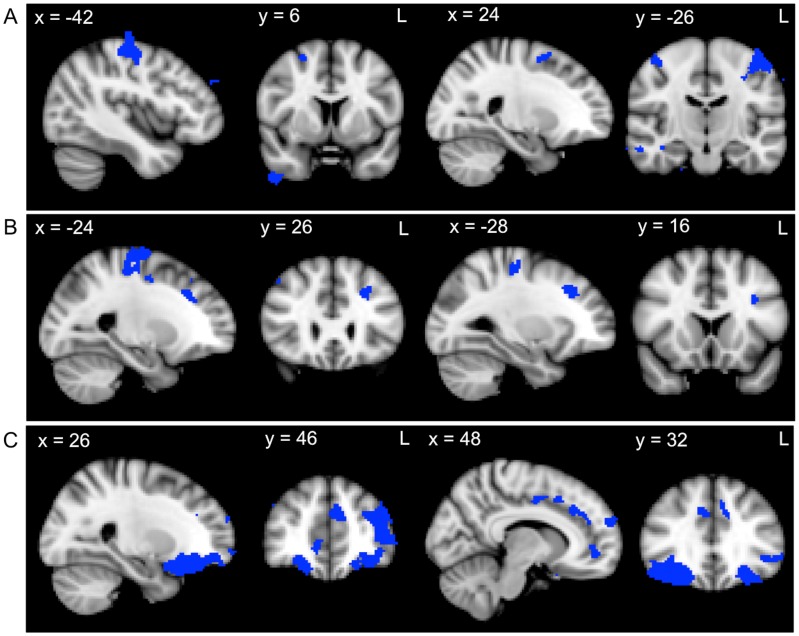
Grey matter atrophy correlates for recall, DLPFC task and VMPFC task performance within the AD group. VBM analyses showing brain regions in which grey matter intensity correlates with the A) recall composite, B) DLPFC task composite and C) VMPFC task composite in AD patients. Clusters are overlaid on the MNI standard brain. Coloured voxels show regions that were significant in the analyses for *p*<.05 FDR corrected and a cluster threshold of 20 contiguous voxels.

In AD patients, recall measures were correlated with atrophy in the pre- and post-central gyri, middle temporal gyrus, supplementary motor cortex, middle and superior frontal gyrus, temporal pole and frontal pole (Figure 3A, Table 4). Whereas the DLPFC task composite scores were related to atrophy in the precentral gyrus and inferior, middle and superior frontal gyri (Figure 3B, Table 4), the VMPFC task composite was associated with atrophy in the orbitofrontal cortex, frontal pole, supplementary motor cortex, paracingulate gyrus and cingulate gyrus (Figure 3C, Table 4).

**Table 4:** Voxel-based morphometry results showing regions of significant grey matter intensity decrease that covary with composite scores within the AD group.

		MNI Coordinates				
Regions	Hemisphere (L/R/B)	X	Y	Z	Numberof voxels	T-score(peak voxel)
***Recall***						
Precentral gyrus	L	−42	−14	46	559	3.11
Precentral gyrus/postcentral gyrus	R	40	−24	58	182	3.11
Middle temporal gyrus (temporo-occipital part)	R	66	−58	6	120	3.11
Supplementary motor cortex	B	2	0	54	116	3.11
Middle temporal gyrus (posterior)	R	70	−6	−26	108	3.11
Superior frontal gyrus	R	22	−2	52	89	3.11
Temporal pole	R	48	8	−52	69	3.11
Postcentral gyrus	L	−64	−20	38	52	3.11
Frontal pole	L	−10	76	12	44	3.11
Frontal pole	R	60	42	−2	44	3.11
Middle frontal gyrus	L	−52	22	30	42	3.11
Frontal pole	R	44	60	−18	35	3.11
Frontal pole	L	−16	62	38	33	3.11
Middle frontal gyrus	R	32	24	26	33	3.11
Supramarginal gyrus/Middle temporal gyrus (temporo-occipital part)	L	−52	−50	12	32	3.11
***DLPFC tasks***						
Precentral gyrus/Superior frontal gyrus	L	−24	−28	52	480	3.11
Middle frontal gyrus	L	−28	24	38	113	2.83
Precentral gyrus	R	12	−16	72	27	2.55
Precentral gyrus	R	22	−16	64	26	2.83
Superior frontal gyrus	L	−18	−6	56	26	2.67
Inferior frontal gyrus	L	−32	14	26	23	3.11
***VMPFC tasks***						
Orbitofrontal cortex/Frontal pole	L	−24	22	−26	2130	3.11
Orbitofrontal cortex/Frontal pole	R	24	20	−26	2101	3.11
Paracingulate gyrus/Cingulate gyrus	B	−12	50	18	621	3.11
Frontal pole	R	12	62	22	124	3.11
Frontal pole	R	38	56	10	63	3.11
Paracingulate gyrus	R	12	48	−4	51	2.55
Frontal pole	L	−22	68	−2	47	2.67
Supplementary motor cortex	R	10	2	46	41	2.83
Frontal pole	L	−4	70	2	31	2.67
Paracingulate gyrus	R	12	16	44	26	2.67

Results FDR corrected at *p*<.05; only clusters with at least 20 contiguous voxels included.

#### Prefrontal contributions to recall composite scores

A partial correlation analysis further explored whether damage to prefrontal regions could have explained the significant correlations with the recall composite score. In bvFTD patients, both DLPFC and VMPFC regions still correlated significantly (*p’s*<.01) with the recall composite score when temporal lobe atrophy was taken into account. In AD patients however, only DLPFC regions remained significantly correlated (*p*<.05) with the recall composite score once temporal lobe atrophy was taken into account.

#### Overlap in recall, DLPFC and VMPFC task atrophy covariate regions

**Figure 4:**
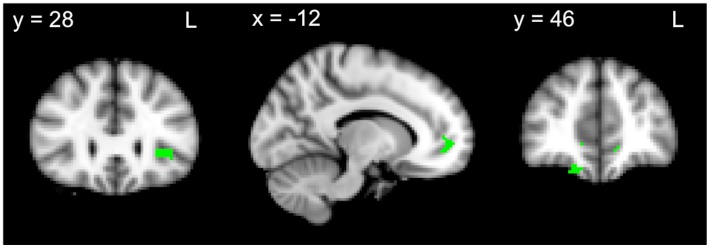
Overlapping regions of grey matter atrophy for the recall and VMPFC task composites across all participants. VBM analyses showing overlap in brain regions in which grey matter intensity correlates with recall and VMPFC task composites across all participants. Coloured voxels show regions that were significant in the analyses for *p*<.05 FDR corrected and a cluster threshold of 20 contiguous voxels.

Finally, we explored whether atrophy covariates of the memory recall composite overlapped with the atrophy covariates of the DLPFC task and VMPFC task composites. For all participants combined, atrophy patterns showed significant overlap between the recall and VMPFC task measures in the orbitofrontal cortex/insular cortex, paracingulate gyrus and frontal pole (Figure 4 Table 5). Although a small region of atrophy correlating with both recall and DLPFC task composites was identified in the inferior frontal gyrus, this failed to reach statistical significance (Table 5). Within bvFTD patients, no regions of overlap were identified for the recall and DLPFC task composites. Within AD patients however, a significant region of overlap for recall and DLPFC task composites was identified in the precentral gyrus (peak voxel: X = −36, Y = −24, Z  = 58). While small regions of overlap for recall and VMPFC task composites were identified within each patient group, these failed to reach statistical significance.

**Table 5:** Voxel-based morphometry results showing regions of significant grey matter intensity decrease that correlate with recall performance and overlap with those which correlate with DLPFC or VMPFC task performance (across all groups).

		MNI Coordinates				
Regions	Hemisphere(L/R/B)	X	Y	Z	Number of voxels	T-score (peak voxel)
***Recall and DLPFC task***						
Inferior frontal gyrus	L	−34	30	0	1*	1.67
***Recall and VMPFC task***						
Insular cortex/Orbitofrontal cortex	L	−32	26	2	43	2.49
Paracingulate gyrus	L	−12	52	−4	25	2.15
Frontal pole	R	16	46	−20	21	2.15

All results FDR corrected at *p*<.05; only clusters with at least 20 contiguous voxels included.

* Non-significant.

## Discussion

The current study investigated the PFC contributions to episodic memory recall performance in bvFTD and AD. Behaviourally, our results confirm that episodic memory recall performance is strongly correlated with DLPFC and VMPFC task performances. However, imaging analysis showed that impaired recall performance is associated with divergent patterns of PFC atrophy in bvFTD and AD. Whereas in bvFTD, PFC atrophy correlates for recall encompassed DLPFC, VMPFC and frontal pole regions, only the DLPFC and frontal pole were implicated in AD. Importantly, after controlling for temporal lobe atrophy, both DLPFC and VMPFC regions remained significantly correlated with recall performance in bvFTD, whereas only DLPFC atrophy remained correlated with recall performance in AD.

On a behavioural level, the current findings provide further support to a growing body of evidence, which suggests that bvFTD and AD patients are impaired to a similar degree on standard neuropsychological measures of episodic memory recall [17]–[19], [24]. Successful memory recall is multifaceted, however, and poor performance may be due to the disturbance of different underlying mechanisms. The current study aimed to compare the contribution of DLPFC- and VMPFC-mediated processes to episodic memory recall impairments in bvFTD and AD. Consistent with previous findings [14], [32], [33], our results demonstrate that both patient groups are impaired on standard neuropsychological measures that tap into DLPFC function. This likely reflects the comparable severity of DLPFC atrophy found in both patient groups [35]. In contrast, performance on VMPFC tasks was significantly worse in bvFTD compared to AD patients, consistent with the typical pattern of atrophy evident early in bvFTD [35]–[37]. Correlations between the recall composite and DLPFC or VMPFC task composite scores were significant across all groups, however, this did not reach significance within patient groups, likely due to a lack of statistical power. Another possible explanation is the heterogeneity of DLPFC- and VMPFC-mediated functions targeted by the measures included in our composite scores. The use of composite scores did not allow disentangling specific aspects of these prefrontal functions, which may differentially contribute to recall performance and needs to be addressed in future studies.

Our imaging findings support the notion that episodic memory deficits in bvFTD and AD are mediated by different neural mechanisms. Previous studies have demonstrated that divergent patterns of atrophy and hypometabolism underlie the memory deficits evident in both AD and bvFTD [19], [24]. Whereas medio-parietal and temporal regions are implicated in AD, neural correlates of memory impairment in bvFTD include lateral and medial frontal, frontal-subcortical and anterior temporal regions [19], [24]. Our imaging results support these previous findings by showing that both frontal and temporal regions are correlated with episodic memory recall across patient groups. Whilst previous studies have contrasted the neural correlates of episodic memory using whole brain approaches [19], [24], we sought to further elucidate specific prefrontal contributions to memory recall using a combination of region-of-interest analyses and partial correlations, which showed that divergent patterns of PFC atrophy are associated with recall performance in bvFTD and AD. Crucially, VMPFC regions were implicated in bvFTD only, whereas DLPFC and frontal pole atrophy was correlated with recall performance in both patient groups. These results are in line with earlier findings that these regions are implicated in episodic memory deficits in bvFTD [18], [19], [24]. Furthermore, the prefrontal regions remained significantly correlated with recall performance even after controlling for temporal lobe atrophy. This suggests that DLPFC atrophy independently contributes to the memory impairments in both patient groups, with additional involvement of the VMPFC in bvFTD only.

Previous studies have highlighted the role of the DLPFC in the strategic aspects of episodic memory recall [28], [29]. Together with previous reports that bvFTD and AD patients show comparable levels of DLPFC atrophy [35], our findings suggest that episodic memory deficits in both patient groups are related to the disruption of DLPFC-mediated strategic retrieval processes. Previous studies investigating these processes using conventional measures of executive function (e.g. COWAT and digit span backwards) suggest that impaired performance on these tasks is not specific to bvFTD, with AD patients also showing deficits [33], [67]. Another potential explanation for the DLPFC involvement is that it reflects an inherent bias towards the use of strategic retrieval processes in standard neuropsychological measures of episodic memory recall. It is therefore possible that current measures of episodic memory recall lack sufficient specificity to distinguish between the two patient groups because they target memory processes that require the DLPFC, a region which is similarly affected in bvFTD and AD [35]. By contrast, an association between episodic memory performance and VMPFC integrity was found in bvFTD patients only. The VMPFC has been shown to be involved in various social-executive cognitive processes, including theory of mind [68], self-referential processing and perspective taking [69], emotion processing [70] and inhibition [71]. Not surprisingly, bvFTD patients are known to be impaired on tasks tapping into VMPFC functions [72], [73]. We confirm this notion by showing more impairment on VMPFC-mediated tasks in bvFTD when compared to AD. Findings from our overlap analyses, however, suggest that the atrophy correlates for recall, DLPFC and VMPFC task performance show only minimal similarities. This is likely due to the heterogeneity of measures included within our prefrontal composite scores. It is also possible that these measures quantify individual prefrontal mechanisms rather than their contributions to episodic memory recall per se.

Taken together, our results indicate that performance on measures tapping into VMPFC function can distinguish between bvFTD and AD patients, and that there is greater involvement of VMPFC regions in episodic memory recall in bvFTD. It may therefore be worthwhile employing episodic memory tasks that tap into VMPFC functions. One potential approach could involve the self-reference effect on memory, where information that is evaluated in reference to one’s self is better remembered than information that is evaluated external to one’s self [74]. For example, items that have been subjectively rated for pleasantness are better remembered than items rated for similarity to background colour [41]. Importantly, retrieval of self-relevant information is associated with the integrity [75] and activity [76] of the medial PFC (MPFC). Similarly, Leshikar and Duarte [41] demonstrated that activation of the MPFC during the self-referential encoding of information is predictive of subsequent accuracy in source memory retrieval. The incorporation of such measures may therefore provide important insights into the role of VMPFC regions in episodic memory retrieval in bvFTD.

From a clinical perspective, identifying differences in the underlying mechanisms of episodic memory deficits in bvFTD and AD may support differential diagnosis of these cohorts. Our findings confirm that performance on verbal and visual episodic memory recall tasks do not reliably distinguish between bvFTD and AD patients. Given that memory impairment remains an exclusion criterion, this may limit the sensitivity of current diagnostic criteria for bvFTD [3]. One potential reason for the limited sensitivity of current neuropsychological measures of episodic memory recall is their reliance on DLPFC-mediated strategic retrieval processes. Importantly, our findings also demonstrate that both bvFTD and AD patients are impaired on executive measures that tap into DLPFC function. While current diagnostic criteria for bvFTD describes a predominantly dysexecutive profile [3], revised criteria for AD also allow for nonamnestic presentations, with prominent executive dysfunction [77]. As such, the inclusion of measures that tap into VMPFC-mediated social-executive functions appears to be a promising approach. Accordingly, previous studies that have compared DLPFC- and VMPFC-mediated executive functions in low and high functioning bvFTD patients showed that VMPFC-mediated functions were affected in both the low and high functioning patient groups, whereas impairments on DLPFC-mediated tasks were evident in low functioning patients only [72], [73]. This supports the notion that VMPFC- rather than DLPFC-mediated tasks are more sensitive to the earliest neuropathological changes in bvFTD, which occur in the VMPFC before progressing to the DLPFC [37], [72], [73]. Therefore, incorporation of these measures into standard clinical assessments would likely contribute to earlier diagnosis and treatment. Nonetheless further exploration of the divergent neural mechanisms underlying episodic memory deficits in bvFTD and AD is warranted.

There are a number of caveats to consider. Firstly, despite the significant hippocampal atrophy identified in AD patients compared to controls, hippocampal atrophy correlates for recall performance failed to meet our criteria for statistical significance. While it is possible that regions not included in our frontal and temporal lobe mask show stronger correlations with recall performance in AD, this finding is difficult to explain. Similarly, the involvement of pre- and postcentral gyrus and supplementary motor cortex atrophy in recall performance in AD was an unexpected finding. However, precentral gyrus atrophy [35] and correlations between recall performance and pre- and post-central gyrus atrophy [24] have previously been reported. There is also some evidence to suggest that these motor and premotor regions are involved in the maintenance of verbal or visual information in working memory [78], [79]. Given that atrophy in these regions were also correlated with DLPFC task performance, it is likely that deficits in working memory contribute to poor episodic memory recall. Nevertheless, replication of our results using specific measures of working memory rather than a composite of DLPFC-mediated tasks represents an important area of future inquiry. Finally, the impact of prefrontal grey matter atrophy on underlying white matter tracts remains to be elucidated. Given that the hippocampus shares reciprocal connections with both the DLPFC and VMPFC [38], [80], future studies should explore the prefrontal white matter contributions to episodic memory deficits.

A number of methodological issues warrant discussion. Firstly, neuropathological confirmation of the patients’ clinical diagnoses were not available, given that the majority of our sample had not yet come to autopsy. Although we cannot exclude the possibility that some bvFTD patients had underlying AD pathology, our findings are consistent with previous reports of memory impairment in pathologically confirmed bvFTD cases [8], [9], [17]. Secondly, the patient groups were matched on the FRS but not CDR sum of boxes. This is unsurprising, given that the two measures emphasize different aspects of disease severity. The FRS encompasses everyday cognition and functional dependence, whereas the CDR is more cognitive and memory oriented. Nonetheless, the use of dementia-specific clinical disease severity staging tools to compare across dementia types remains controversial. Furthermore, the use of composite measures to probe episodic memory recall and DLPFC- or VMPFC-mediated functions did not allow the dissection of specific aspects of these functions. Heterogeneity in memory recall test administration should also be taken into account, given that the RAVLT involves incremental and explicit learning, whereas the RCF test is based on one-trial incidental learning. Nonetheless, our findings help elucidate the PFC correlates of general episodic memory recall dysfunction in different neurodegenerative conditions.

To our knowledge, this is the first study to explore specifically the prefrontal neural correlates of episodic memory recall deficits in bvFTD and AD. The behavioural results of our study call into question the specificity of memory recall impairment in discriminating between the neurodegenerative conditions. Taken together, our behavioural and imaging findings suggest that although divergent prefrontal mechanisms may underlie episodic memory deficits in bvFTD and AD, these are not adequately captured by existing neuropsychological measures. Thus, development of tests that specifically target VMPFC contributions to memory recall would likely further elucidate differences in the nature of memory impairment in bvFTD and AD.

## Supporting Information

Figure S1 **Grey matter atrophy comparisons between groups.** VBM analyses showing brain areas of decreased grey matter intensity in A) bvFTD patients in comparison with Controls, B) AD patients in comparison with Controls, C) bvFTD patients in comparison with AD patients, and D) AD patients in comparison with bvFTD patients. Patient and control group comparisons corrected for multiple comparisons (FWE) with voxel-based thresholding at *p*<.05. Comparisons between patient groups corrected for multiple comparisons (FWE) with threshold-free cluster enhancement at *p*<.025. Clusters are overlaid on the MNI standard brain.
(TIF)

Table S1 **Mean raw scores for bvFTD, AD patients and controls on neuropsychological measures.**
(DOCX)
