# Supplementary figures and images for: Contrasting Prefrontal Cortex Contributions to Episodic Memory Dysfunction in Behavioural Variant Frontotemporal Dementia and Alzheimer’s Disease
Source: PLoS One. 2014 Feb 4;9(2):e87778. doi: 10.1371/journal.pone.0087778 (PMC3913699; doi:10.1371/journal.pone.0087778)

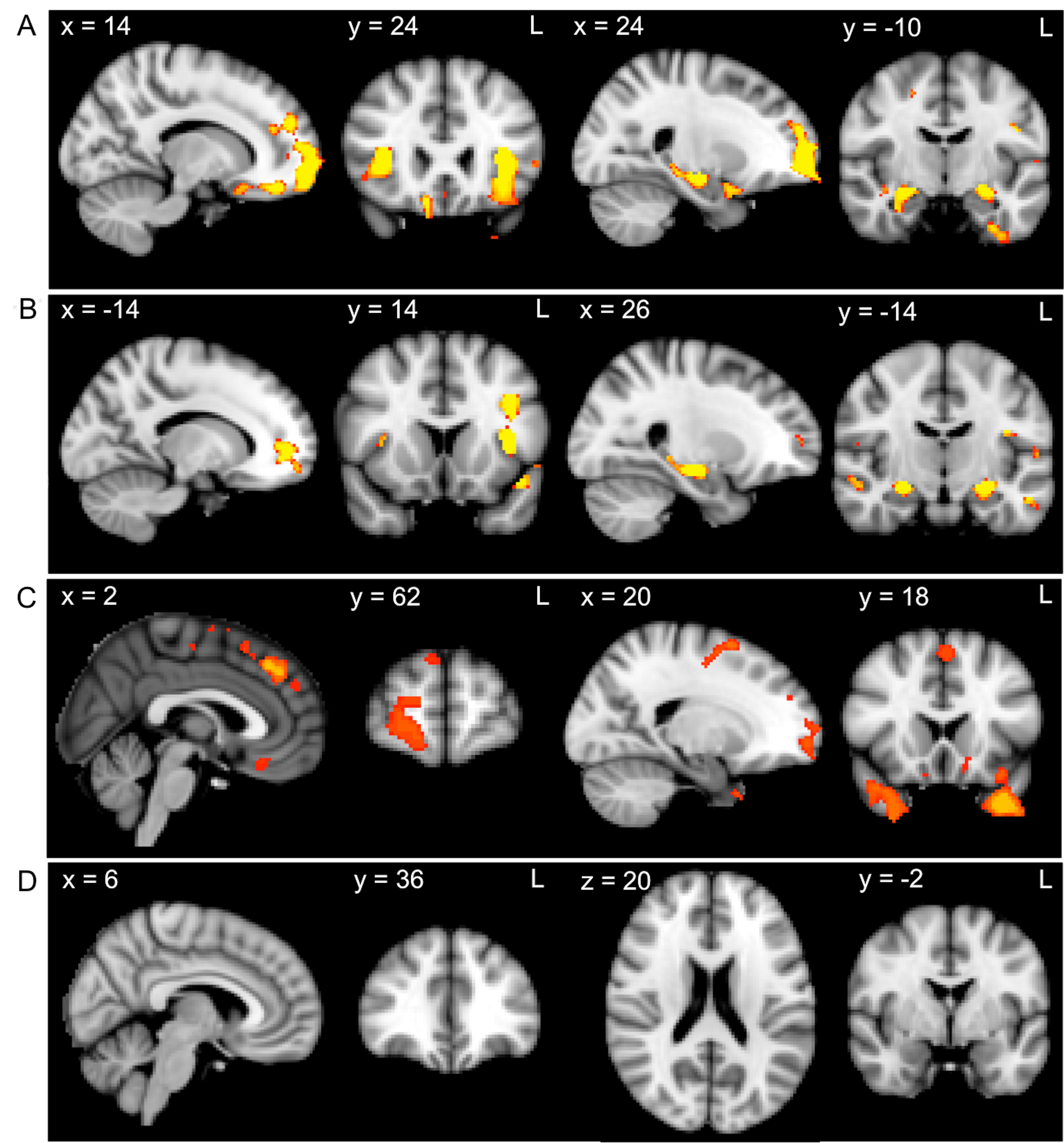

Supplement: Figure S1 — Grey matter atrophy comparisons between groups. VBM analyses showing brain areas of decreased grey matter intensity in A) bvFTD patients in comparison with Controls, B) AD patients in comparison with Controls, C) bvFTD patients in comparison with AD patients, and D) AD patients in comparison with bvFTD patients. Patient and control group comparisons corrected for multiple comparisons (FWE) with voxel-based thresholding at p<.05. Comparisons between patient groups corrected for multiple comparisons (FWE) with threshold-free cluster enhancement at p<.025. Clusters are overlaid on the MNI standard brain. (TIF) [file pone.0087778.s001.tif]
