# Supplementary material for: Contrasting Prefrontal Cortex Contributions to Episodic Memory Dysfunction in Behavioural Variant Frontotemporal Dementia and Alzheimer’s Disease
Source: PLoS One. 2014 Feb 4;9(2):e87778. doi: 10.1371/journal.pone.0087778 (PMC3913699; doi:10.1371/journal.pone.0087778)
Supplement: Table S1 — Mean raw scores for bvFTD, AD patients and controls on neuropsychological measures. (DOCX) [file pone.0087778.s002.docx]

*Table S1. Mean raw scores for bvFTD, AD patients and controls on neuropsychological measures ^a^*

|  | ***Control*** | ***bvFTD*** | ***AD*** | ***Group effect*** | ***bvFTD vs Control*** | ***AD vs Control*** | ***bvFTD vs AD*** |
| --- | --- | --- | --- | --- | --- | --- | --- |
| **RAVLT A6 recall [15]** | 10.11 (2.71) | 3.4 (3.07) | 2.44 (2.92) | *** | *** | *** | n.s. |
| **RCF 3 min. recall [36]** | 17.35 (5.16) | 6.55 (5.69) | 3.13 (3.69) | *** | *** | *** | n.s. |
| **Digits Backwards raw score [14]** | 7.94 (2.63) | 4.32 (2.21) | 4.13 (1.75) | *** | *** | *** | n.s. |
| **FAS Verbal Fluency total correct** | 43.94 (12.05) | 22.19 (11.55) | 27.03 (11.47) | *** | *** | *** | n.s. |
| **Brixton total error [54]** | 16.09 (6.23) | 25.89 (13.00) | 26.25 (8.78) | ** | * | ** | n.s. |
| **Hayling total AB score [128]** | 1.2 (1.76) | 37.64 (28.12) | 16.19 (17.80) | *** | *** | *** | ** |
| **Iowa Gambling Task modified total net score (deck D- deck A)** | 27.72 (15.37) | 4.8 (18.67) | 8.33 (18.3) | *** | *** | ** | n.s. |
| **TASIT total correct [28]** | 23.87 (2.05) | 15.61 (5.07) | 18.10 (4.43) | *** | *** | *** | n.s. |

^a^Standard deviations in parentheses, maximum score for tests shown in brackets.

**p*< .05, ***p*<.01, ****p* <.001, n.s = non-significant
